# Could it Be Diagnosed Earlier? A Retrospective Analysis of Lung Cancer

**DOI:** 10.5152/eurasianjmed.2025.25893

**Published:** 2025-08-08

**Authors:** Fatma Tokgöz Akyıl, Hülya Abalı, Sinem Nedime Sökücü, Nurdan Şimşek Veske, Metin Sucu, Çiğdem Sabancı, Sida Gösterici, Sedat Altın

**Affiliations:** 1 Department of Chest Diseases, Yedikule Chest Diseases and Thoracic Surgery Training and Research Hospital, İstanbul, Türkiye; 2 Department of Radiology, Istanbul Florence Nightingale Hospital, İstanbul, Türkiye

**Keywords:** Chest X-ray, malignancy, thorax computed tomography

## Abstract

**Background:**

Early diagnosis is one of the most critical factors influencing the prognosis of lung cancer. This study aims to investigate radiological diagnostic delays and their underlying causes in lung cancer.

**Methods:**

The previous radiological images of newly diagnosed lung cancer patients were retrospectively reviewed by a multidisciplinary team consisting of 2 chest physicians and 1 radiologist. Radiological abnormalities were identified, and potential factors contributing to delayed diagnosis were analyzed.

**Results:**

Among 100 patients, 76 had prior thoracic imaging. In 22 of these, lesions had been previously identified and patients informed about the potential risk of malignancy, but further evaluation was declined. Retrospective review revealed unrecognized radiological abnormalities in 30 patients—12 on chest X-rays and 18 on computed tomography (CT) scans. In 7 cases, the lesions had been documented in the CT reports. Lesions located in peri-hilar, tracheobronchial, and paravertebral regions, as well as those originating from areas of lung sequelae, were among the most common factors associated with later diagnosis. Diagnostic delays were more common in cases of adenocarcinoma.

**Conclusion:**

A considerable proportion of lung cancer cases might have been detected earlier. Increased awareness of specific radiological features and careful co-evaluation of both imaging reports and the images themselves may enhance earlier detection in lung cancer.

**Main Points**

- Lung cancer diagnosis may be delayed despite prior radiological imaging.
- All imaging studies and corresponding radiology reports should be carefully reviewed, regardless of the requesting physician’s specialty.
- Particular attention should be given to lesions located in lung sequelae areas, as well as perihilar and central airways.

## Introduction

Early-stage diagnosis is one of the most critical factors influencing the prognosis of lung cancer.^1^^,^^2^ Stage progression due to delays attributable either to the patient or the physician is well acknowledged in practice.^3^ Radiologic imaging plays a central role in diagnosis; however, abnormalities may be overlooked and only identified later during retrospective review of prior studies. Lung cancers that are not detected on initial imaging but are recognized in hindsight represent a persistent and controversial issue. Despite efforts to reduce diagnostic errors, eliminating them entirely, even among the most experienced thoracic radiologists, is unlikely. Although such errors are multifactorial and may be unavoidable to some extent, strategies to minimize them merit further investigation.

In the United States, bronchial and lung malignancies are the sixth most common cause of medico-legal claims and the second most litigated condition involving radiologists.^4^ Numerous studies have addressed the technical adequacy in lung imaging, interpretation under optimal conditions, differentiation between malignant and benign lesions, and approaches to minimize diagnostic errors. These studies have evaluated both chest radiography^5^^-^^10^ and thoracic computed tomography (CT) imaging.^11^^-^^15^ Reports indicate that more than half of newly diagnosed lung cancer patients had radiological abnormalities detectable on prior imaging performed as part of cancer screening programs.^16^

The underlying reasons for unrecognized lesions can be categorized into observer/interpreter-related factors, tumor characteristics, and/or technical errors.^17^ Diagnostic accuracy is influenced by several observer/interpreter-related variables, including medical experience, specialty, fatigue, time allocated for image review, and availability of clinical history. Additionally, lesion-specific features such as size, density, and margins, as well as the imaging modality used, affect the likelihood of detection errors.^8^^,^^17^

Although thoracic CT has reduced the likelihood of missed lesions, the risk of diagnostic oversight persists. In this context, the importance of tumor location and comparison with prior imaging studies is frequently emphasized. Such comparisons, between the initial imaging where the lesion was missed and the subsequent imaging that led to diagnosis, allow for chronological re-evaluation. This may contribute both to improved prognosis and to a better understanding of diagnostic delays. Moreover, radiologists’ workload has been identified as a significant factor affecting the accuracy of lesion interpretation.^14^

The aim of this study is to evaluate the frequency and potential causes of unrecognized lung lesions, subsequently diagnosed as lung cancer, that might have been detected on prior imaging.

## Materials and Methods

Patients who were diagnosed with lung cancer between December 1, 2023, and June 1, 2024, were identified. Previous imaging studies were retrospectively reviewed. The chest X-rays and/or thoracic CT scans that established the diagnosis were compared with imaging obtained within the preceding 5 years. Earlier imaging was retrieved by screening the Picture Archiving and Communication System (PACS), which provides automated access to hospital-based radiologic records.

The study was approved by the Ethics Committee and Scientific Research Committee of Yedikule Chest Diseases and Thoracic Surgery Training and Research Hospital (Approval No: 359-3 Date: 8.11.2023). Informed consent was obtained from the patients participating in the study to review their previous imaging studies.

### Recorded Parameters

The following parameters were recorded: patient demographics; dates of subsequent diagnostic procedures and pathological diagnosis; primary tumor type and size; and positron emission tomography (PET-CT) findings. In addition, prior lung imaging studies performed within the past 5 years, including examination dates and corresponding radiology reports, were documented.

### Study Design

Based on the recorded data, patients were initially categorized according to the availability of previous imaging studies. For patients with prior radiological imaging, all relevant images were reviewed and further classified based on the presence or absence of missed radiological findings. These images were independently assessed by a multidisciplinary team consisting of 2 chest physicians and 1 radiologist. A lesion was classified as “unrecognized” on previous images if at least 2 of the evaluators identified it as such. If at least 2 physicians considered that the lesion was not present on the earlier images, the images were classified as showing “no radiological abnormalities.” During the evaluations, the 3 experts reached unanimous agreement in 70 of the 76 cases. In the remaining 6 cases, a majority decision was made, with 2 experts (1 radiologist and 1 chest physician) agreeing, while the other chest physician dissented.

Patient-related delay: Lesions that had been previously recognized but where patients were lost to follow-up despite being informed of the potential malignancy risk were classified as cases with patient-related diagnostic delays.

Unrecognized lesions: Lesions with radiological findings at the tumor site that were present on earlier imaging but not identified at the time—according to the evaluation of a multidisciplinary team—were classified as unrecognized lesions. These lesions were further subclassified based on whether they had been overlooked on chest X-rays or CT scans.

After identifying the unrecognized lesions, the 2 chest physicians and the radiologist assessed possible factors contributing to their initial oversight.

Final etiologies were categorized as follows:

1. Perihilar lesions: lesions at or around the hilar region.
2. Changes in pulmonary sequelae: Progression or changes in pre-existing sequelae lesions.
3. Small and/or low-density nodules: Nodules visible but difficult to clearly identify due to small size or low attenuation.
4. Subclavicular lesion (chest X-ray): Lesions situated below the clavicle.
5. Imaging for preoperative evaluation: Lesions identified on imaging performed for preoperative purposes.
6. Images obtained in the intensive care unit: Lesions observed on imaging studies conducted in the ICU.
7. COVID-19 era imaging: Imaging performed during COVID-19 pandemic.
8. Imaging after blunt chest trauma: Imaging conducted following blunt chest trauma.
9. Ground glass opacity in subsequent imagings: Ground-glass opacity showing no regression and slow progression over more than 2 years.
10. Lesion with cystic appearence: Lesions demonstrating cystic characteristics.
11. Incidental finding on lumbar CT: incidental pulmonary nodules detected on lumbar CT scans.
12. Tracheobronchial lesions: Lesions involving the trachea and/or bronchi.
13. Paravertebral lesion: Lesions located adjacent to the vertebrae.
14. Unknown etiology: The unrecognized lesions could not be attributed to any identifiable anatomical or etiological factors. A comparative analysis of tumor types and primary lesion characteristics was performed between cases with and without unrecognized lesions, excluding those with patient-related diagnostic delays to minimize bias.

### Statistical Analysis

Data were recorded and analyzed using the statistical software package SPSS for Windows 16.0 (SPSS Inc., Chicago, IL, USA). Data are presented as mean ± SD and percentages. Chi-square tests and Student’s *t*-tests were used for inter-group comparisons. A *P*-value of < .05 was considered statistically significant.

## Results

The study included 100 patients with a mean age 64 ± 10 (range: 28-89) and 81 were male. Thirty-five patients had no comorbidities. The additional diseases were obstructive pulmonary disease (n = 22), coronary artery disease (n = 20), hypertension (n = 16), diabetes mellitus (n = 13), congestive heart failure (n = 7), cerebrovascular disease (n = 4), and other (n = 7). Ever smoker patient rate was 76%. Five patients were never-smokers, while 69 were current smokers. The mean smoking history was 43.6 ± 25.5 pack-years (range: 5-160).

No prior thoracic imaging records were available for 24 patients. In another 24 patients, previous imaging studies revealed no abnormal findings.

In the remaining 52 patients, prior imaging demonstrated lesions. Among these, 22 patients experienced delays attributed to patient-related factors. Although these patients had been informed of the potential malignancy risk upon lesion detection, 16 declined further clinical evaluation at the time, and 6 did not attend scheduled follow-ups. This non-compliance resulted in a diagnostic delay, with a mean duration of 9.5 ± 7 months (range: 3-30 months).

**Figure 1.**
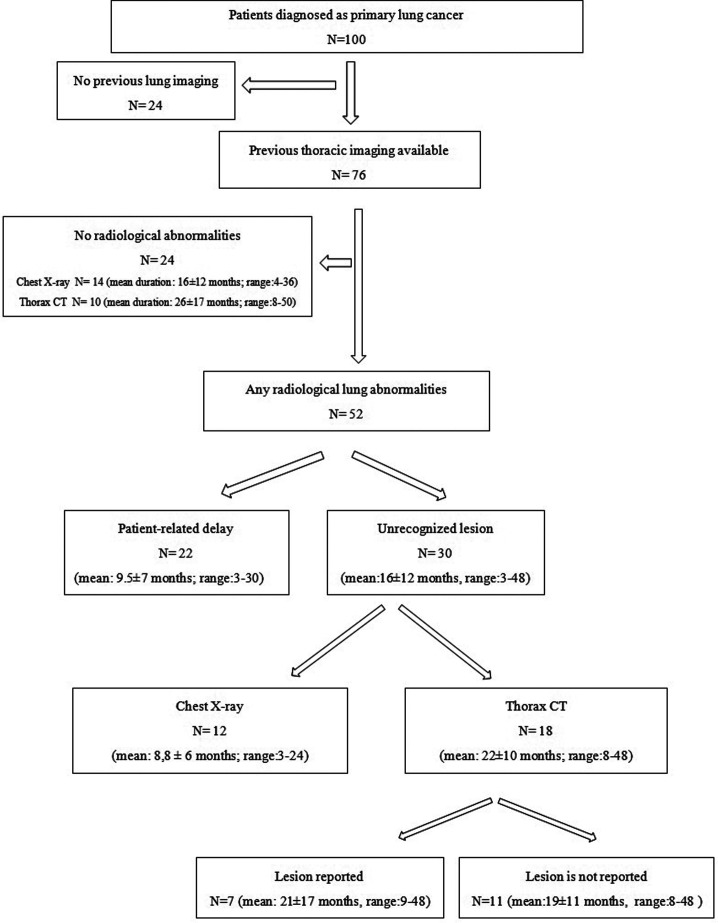
Flowchart of the patients based on previous radiological imaging studies.

In 30 patients, prior imaging had been performed, and lesions were retrospectively classified as “unrecognized.” The diagnostic delay related to these unrecognized lesions had a mean duration of 16 ± 12 months (range: 3-48 months; median: 12 months). The imaging modalities involved included chest X-rays in 12 patients and CT scans in 18 patients (Figure 1).

The overall median diagnostic delay for chest X-rays was 8.8 months (range: 3-24 months). Delayed diagnoses were most frequently associated with imaging studies performed in emergency departments (n = 6; 50%), followed by internal medicine (n = 4), chest diseases (n = 1), and urology departments (n = 1). Lesions located in lung sequelae areas and perihilar regions were the most common contributors to unrecognized lesions (Table 1).

**Figure 2.**
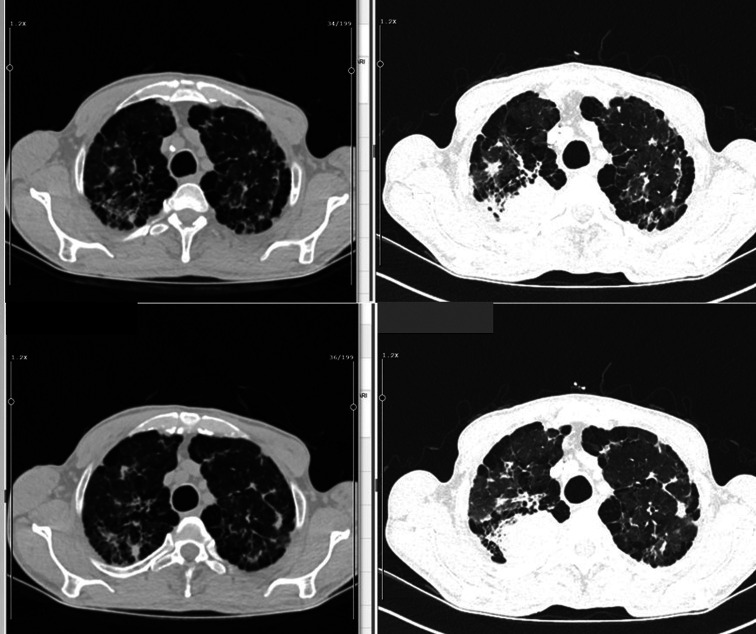
(A) Chest computed tomography taken 23 months prior (left side) (B) at the time of diagnosis (right side).

**Figure 3.**
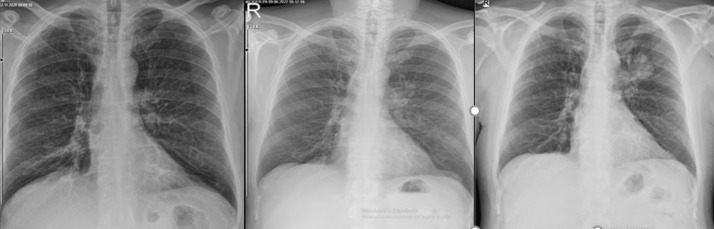
Sequential chest X-rays of a patient taken 3 years prior (left), 15 months prior (middle), and at the time of diagnosis (right).

**Figure 4.**
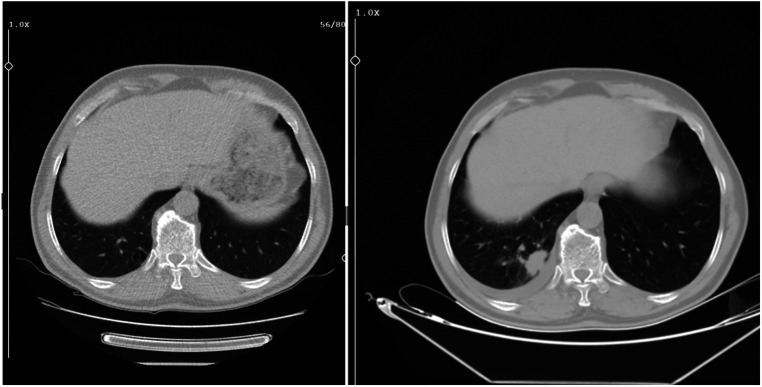
Thorax computed tomography taken 2 years prior (left) and at the time of diagnosis (right).

The mean diagnosis delay time of unrecognized lesions in CT was 22 months (range: 8-48 months). These prior images were taken in the emergency department (n = 9, 50%), followed by internal medicine (n = 2), chest diseases (n = 2), neurology (n = 1), intensive care unit (n = 1), physical therapy and rehabilitation (n = 1), and other (n = 2) (Table 1) (Figures 2-4). In 7 cases, the lesions had been documented in CT reports; however, either the patient was not informed afterward or did not attend follow-up visits to discuss the CT reports.

The final pathological diagnoses were as follows: small-cell lung cancer in 13 patients, mesenchymal tumors in 3 patients, and non-small-cell lung cancer (NSCLC) in 84 patients (adenocarcinoma: n = 38, squamous cell carcinoma: n = 30, unspecified NSCLC: n = 16).

No diagnostic delay were observed in patients with small-cell lung cancer or mesenchymal tumors. The 30 unrecognized lung lesions were distributed as follows: adenocarcinoma (n = 18), squamous cell carcinoma (n = 9), and unclassified NSCLC (n = 3). Diagnostic delays were significantly more frequent in cases of adenocarcinoma (*P* = .01).

Among the 77 patients for whom tumor staging was available at the time of diagnosis, tumor size did not differ significantly between the groups with recognized and unrecognized lesions (5.5 ± 2.7 cm vs. 5.7 ± 2.6 cm, respectively; *P* = .48).

## Discussion

The present study, conducted at a specialized center for chest diseases, highlights that delays in lung cancer diagnosis can occur even when imaging has been performed. Investigation of the underlying causes of these delays highlights 2 critical issues. First, the increasing burden on healthcare services may exceed the cognitive capacity of healthcare professionals, leading to potential errors. Second, there is a need for physicians to adopt new perspectives when interpreting radiological images and integrating these findings with clinical information. Relying solely on conventional approaches may perpetuate diagnostic errors. A more manageable workload, combined with a renewed focus on identifying potential lesions to avoid misdiagnosis, could reduce both the incidence of diagnostic errors and the costs associated with delayed diagnoses. It is reported that medico-legal cases related to lung cancer predominantly involve chest X-rays (90%), followed by thoracic CT scans (5%), with other imaging modalities accounting for the remaining 5%.^15^ Interestingly, in the present study, most cases of unrecognized lesions were detected retrospectively on CT scans rather than chest X-rays. This discrepancy may be attributed to the increased accessibility and more frequent use of CT imaging at various stages of clinical evaluation. However, the increased utilization of CT scans may also contribute to a higher workload for radiologists, potentially increasing the likelihood of diagnostic errors. In this context, artificial intelligence (AI)-based programs offer promising solutions to enhance diagnostic accuracy and reduce interpretation time. The authors believe that further comparative studies are needed in the authors’ country to explore the potential role of AI in mitigating diagnostic delays.

In a lung cancer screening program, Xu et al^16^ evaluated 104 newly diagnosed lung cancer patients and found that 80 patients (77%) had abnormalities on imaging performed 1 year prior. They reported that in 18% of these cases, the initially missed imaging had revealed stage I cancer, which later progressed to more advanced stages due to diagnostic delays. The tumor doubling time in these patients was calculated to be 108 days. In contrast, patients whose prior imaging was entirely normal exhibited significantly faster tumor growth, with a doubling time of 43 days, classifying these tumors as aggressive.^16^

Although detailed tumor staging was not performed in this study, tumor size was similar between groups with and without diagnostic delays. This may be explained by longer tumor doubling times in the group with missed lesions. Consistent with the findings of Xu et al,^16^ the authors observed that the lower likelihood of missed diagnoses in small-cell lung cancer is likely due to its aggressive nature. This suggests that in cases where tumor-related factors contribute less to detection challenges, physicians need to be even more attentive to avoid overlooking lesions.

Lesions may be missed on imaging due to various types of errors, most commonly attributed to observer-related factors. Interpretation errors are typically categorized by frequency as scanning errors, recognition errors, and decision-making errors. Scanning errors may result from limited medical experience, failure to adopt a systematic approach during image evaluation, or inadequate visual fixation. In this context, studies suggest that approximately 300 fixations are required to detect a lesion, whereas in clinical practice, physicians may perform only 80-100 fixations. Recognition errors can be influenced by factors such as the working conditions of the interpreting physician or radiologist, fatigue and exhaustion levels, and insufficient clinical history detail. Decision-making errors are primarily associated with the clinician’s level of experience. Once a diagnostic decision is made, any delays in diagnosis may become irreversible or only rarely corrected.^8^^,^^17^, ^18^

In light of these factors, the authors re-evaluated their own study population. Four cases of sequela-based cancers may be attributable to the working conditions of physicians. Patients with sequela lesions often have frequent hospital admissions, which may limit the time and attention allocated for comprehensive evaluations. Under such circumstances, comparing previous and recent images becomes particularly challenging, especially in settings characterized by high workload and time constraints. For example, in 1 patient imaged who underwent imaging due to trauma, a nodular lesion was not reported, potentially due to the focus being on trauma-related findings. In 8 patients, no specific cause could be identified for the missed lesions; however, the authors believe that factors such as physician or radiologist fatigue or insufficient clinical history are likely contributors. Based on these considerations, studies investigating whether the number of patients or images evaluated by physicians affects reporting quality or clinical decision-making may provide valuable insights for improving healthcare practices in the authors’ country.

In terms of tumor-related factors, lesion size and conspicuity remain critical. For instance, only 29% of lesions smaller than 1 cm are typically identified, while the rate of missed lesions is reported to be 28% for those measuring between 1-3 cm. The rate decreases to 12% when the lesion size reaches 3-4 cm.^8^ Similarly, factors such as lesion density, border clarity, tumor histology, and anatomical location are also significant in cases of diagnostic oversight. Additionally, technical aspects such as imaging modality, patient positioning, and the lack of lateral chest X-rays can further contribute to interpretive errors. Several interpretation strategies are recommended to minimize such errors: allocating at least 30 seconds to review each image, adopting a systematic approach, paying particular attention to blind spots, requesting lateral projections when suspicion persists, comparing with previous imaging studies, and integrating clinical history into the radiological interpretation.^17^ In this study, the authors confirmed that the likelihood of overlooking lesions increases when nodules are of low density or small in size. Furthermore, lesions located in subclavicular, and paravertebral regions, were associated with diagnostic delays. Careful attention to these tumor characteristics and anatomical locations is crucial for minimizing missed detections and delays in diagnosis.

In thoracic CT evaluations, particular attention should be paid to tumor location and endobronchial involvement.^14^ In 1 study, lesions were present but unreported in prior thoracic CT scans in 7 out of 22 newly diagnosed patients. The reasons for these diagnostic errors included lesion size smaller than 7 mm, juxtavascular localization, and tumors arising from pre-existing pulmonary sequelae.^19^ An analysis of CT scans conducted within the framework of the NELSON screening program identified the primary factors contributing to missed lesions as observer errors, definition errors, and identification errors. These errors were frequently associated with endobronchial and juxta-pleural locations, proximity to bullous structures, and honeycombing patterns.^20^ Furthermore, the heavy workload of radiologists—who must review numerous scans within limited timeframes—has been reported as a factor increasing the risk of interpretive errors.^21^ Additionally, the use of low-dose thoracic CT may result in overlooked lesions when tumor borders are indistinct.^22^

In this study, lesions located in central airways were found to be prone to oversight, as observed in 3 cases. This leads us to recommend meticulous evaluation of paravertebral and central airways and bronchi regions in particular. Reducing workload through the implementation of AI and computer-assisted reporting systems offers an undeniable opportunity. Concrete proposals exist suggesting that AI-driven tools could be integrated into the interpretation process as an additional checkpoint alongside radiologists.^17^

It is also important to note that in many hospitals, CT scan interpretations are outsourced to private third-party services. This outsourcing often limits direct communication between the treating physicians and radiologists, resulting in a lack of shared clinical information and consultation. The authors emphasize that multidisciplinary collaboration between chest specialists and radiologists is essential to minimize diagnostic errors and improve early detection.

Another important finding in this study is that a significant proportion of the imagings were performed in the emergency department. Although imaging is generally undertaken to exclude medical emergencies, and interpretations are primarily focused on urgent conditions, patients often leave assuming no abnormality was detected on their imaging. Furthermore, even if patients are referred to pulmonologists, their chance to schedule an appointment and gain timely access to specialized care is frequently limited. In brief, challenges are present for both physicians and patients.

This study has several limitations. First, it is a retrospective, single-center study, which may limit the generalizability of the findings. Second, complete staging data were not available for all patients, limiting a comprehensive analysis of tumor growth dynamics and staging comparisons. Third, the number of patients with available prior imaging was relatively limited, potentially affecting the statistical power of certain subgroup analyses. On the other hand, the strength of this study is that it was conducted at a tertiary referral center serving a wide geographic area, including both local and out-of-city patients, which enhances the applicability of the findings. Additionally, the study included patients with very recent diagnoses, making the findings highly relevant and up-to-date.

In conclusion, a considerable proportion of lung cancer patients experience diagnostic delays. Increased awareness of specific radiological features, as well as the careful co-evaluation of both imaging reports and the images themselves, may facilitate earlier detection. However, the primary underlying factor for such delays appears to be systemic. The high workload across all medical disciplines—especially in emergency departments—may exceed the cognitive capacity of physicians to accurately synthesize clinical and radiological data. In addition, the outsourcing of radiology reporting can hinder effective communication and collaborative interpretation between clinicians and radiologists. Strengthening communication strategies could help enhance diagnostic accuracy and support a more integrated evaluation process. As medico-legal concerns and the healthcare burden per physician continue to rise, these issues demand attention at the policy level. Without structural changes, the ongoing strain on physicians’ physical, professional, and ethical capacities may remain unsustainable, perpetuating delays in diagnosing a disease where timely intervention is critical for patient survival.

## Figures and Tables

**Table 1.** Possible Underlying Reasons for Unrecognized Lesions Based on Imaging Modality and Reporting Status

	Chest X-rays N (N = 12)	Thorax CT—Reported (N = 7)	Thorax CT—Unreported (N = 11)
Peri-hilar lesions	3		
Changes in pulmonary sequelae	3		1
Small and/or low-density nodules	2		1
Subclavicular lesion (chest X-ray)	1		
Imaging for preoperative evaluation	1		
Images obtained at intensive care unit		1	
COVID-19 era imaging		1	
Imaging performed after blunt chest trauma			1
Ground glass opacity in subsequent imagings		2	
Lesion with cystic appearence			1
Incidental finding on lumbar CT			
Tracheobronchial lesions			3
Paravertebral lesion			1
Unknown etiology	2	3	3

## Data Availability

The data that support the findings of this study are available on request from the corresponding author.
